# Transcatheter Aortic Valve Implantation in an Extremely Tortuous S-Shaped Aorta

**DOI:** 10.1155/2017/2936513

**Published:** 2017-03-02

**Authors:** Shuangbo Liu, Olga Toleva, Amir Ravandi, Zlatko Pozeg, Alan Menkis, Malek Kass

**Affiliations:** ^1^Section of Cardiology, St. Boniface Hospital, University of Manitoba, Winnipeg, MB, Canada; ^2^Section of Cardiac Surgery, St. Boniface Hospital, University of Manitoba, Winnipeg, MB, Canada

## Abstract

Transcatheter aortic valve implantation (TAVI) has emerged as an alternative technique to treating aortic stenosis in patients with high surgical risk. We present a case of a successful transfemoral TAVI in a high-risk patient with an extremely tortuous iliofemoral system and a significant S-type bend in the descending aorta. With careful preprocedure planning and using all the techniques available, TAVI can be performed in the most challenging patients.

## 1. Introduction

Transcatheter aortic valve implantation (TAVI) has emerged as an alternative technique to treating aortic stenosis in patients with high surgical risk [1]. With increased utilization of TAVI, specific cases may pose unique challenges, including complex anatomy. We present a case of a successful transfemoral TAVI in a high-risk patient with an extremely tortuous iliofemoral system and a significant S-type bend in the descending aorta.

## 2. Case Presentation

**Figure 1:**
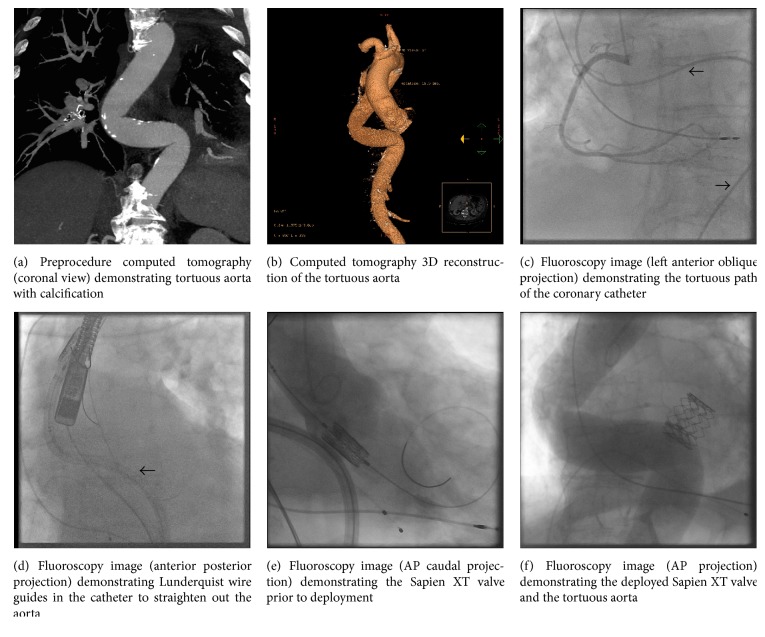
Composite image of a tortuous S-shaped aorta.

An 85-year-old male with atrial fibrillation, diabetes, hypertension, dyslipidemia, previous transient ischemic attack, permanent pacemaker, and symptomatic severe aortic stenosis was deemed very high risk for surgical valve replacement. Transthoracic echocardiography demonstrated severe aortic stenosis (aortic valve area 0.9 cm^2^, mean aortic valve gradient 42 mmHg) and preserved left ventricular ejection fraction and mild pulmonary hypertension. He was referred to, and accepted by the TAVI Heart Team. During the preprocedure workup, he was found to have a very tortuous iliofemoral system, as well as a significant S-type bend in the descending aorta (Figures 1(a)–1(c), Video 1 (see supplementary Video 1 in the Supplementary Materials available online at https://doi.org/10.1155/2017/2936513)). Because of the less robust data with transapical approach [2] and our center's limited expertise with other access methods, we chose to pursue transfemoral TAVI.

On the day of TAVI, bilateral femoral arterial access (8 Fr) was obtained, along with left femoral venous access for transvenous pacing. Via the left femoral arterial sheath, an 8 Fr Amplatz guide catheter was placed into the proximal descending aorta and two Lunderquist (Cook Medical, Bloomington, Indiana) wires were sequentially placed into the aortic arch through this catheter. A pull on the catheter was required to straighten the descending aorta (Figure 1(d), Video 2).

A 20 Fr Sapien e-sheath via the right side was placed in the descending aorta. Valvuloplasty with a 25 mm balloon was performed over an Amplatz wire. A 29 mm Sapien XT valve (Edwards Lifescience, Irvine, California) was advanced into the aorta, which was very technically challenging (Video 3). While trying to manipulate the valve beyond the acute angulation in the aortic root, the ventricular wire was inadvertently pulled. With catheter torquing, a straight glide wire was used to recross and allow the nose cone to cross the native aortic valve. After switching over to a third Lunderquist wire, the valve was correctly aligned (Figure 1(e)) and deployed under burst pacing (Figure 1(f), Video 4). A transthoracic echocardiography demonstrated good valve position and trace aortic insufficiency. At the end of the procedure, an aortogram was performed which did not show any evidence of aortic injury.

## 3. Discussion

TAVI has emerged as an alternative technique to treating aortic stenosis in patients with high surgical risk. Careful preprocedural planning is required for a successful TAVI procedure. The goals of preprocedural planning are to assess the optimal method of access; define anatomic relationships between the aortic valve, root, left ventricle, and coronary ostia; choose the optimal device size; and, lastly, contribute to the procedural plan [3]. Multimodality imaging is required to address these issues, including the use of angiography, multidetector computed tomography (MDCT), echocardiography, and occasionally ultrasound and cardiac magnetic resonance. While echocardiography and angiography are often completed prior to referral to the Heart Team, MDCT also plays a pivotal role in preprocedural planning. MDCT allows assessment of the access site, particularly for transfemoral approach. In our patient, a pre-TAVI workup revealed an extremely tortuous S-shaped aorta and allowed for further planning prior to the procedure.

Tortuous aortas have rarely been described in the literature [4, 5], and we describe a case of a successful transfemoral TAVI being performed through an extremely sigmoid descending aorta using techniques to straighten the vessel. Using all the techniques available, transfemoral TAVI can be performed in the most challenging patients.

## Supplementary Material

Video 1 is a volume rendered video demonstrating the tortuous S-shaped aorta. Video 2 is a fluoroscopy video (anterior posterior projection) showing a pull on the Lunderquist wire guides in the catheter to straighten the S-shaped aorta. Video 3 is a fluoroscopy video (left anterior oblique projection) with the Sapien XT valve system being advanced up the tortuous aorta. Video 4 is a fluoroscopy video (anterior posterior projection) of the aortogram post-TAVI demonstrating no vascular complications.
